# Sphingomyelin metabolism underlies Ras excitability for efficient cell migration and chemotaxis

**DOI:** 10.1247/csf.23045

**Published:** 2023-07-12

**Authors:** Da Young Shin, Hiroaki Takagi, Michio Hiroshima, Satomi Matsuoka, Masahiro Ueda

**Affiliations:** 1 Laboratory of Single Molecule Biology, Department of Biological Sciences, Graduate School of Science, Osaka University, Toyonaka, Osaka, Japan; 2 Laboratory for Cell Signaling Dynamics, Center for Biosystems Dynamics Research, RIKEN, Suita, Osaka, Japan; 3 Department of Physics, School of Medicine, Nara Medical University, Kashihara, Nara, Japan; 4 Laboratory of Single Molecule Biology, Graduate School of Frontier Biosciences, Osaka University, Suita, Osaka, Japan; 5 PRESTO, JST

**Keywords:** cell polarity, cell migration, Ras, excitability, sphingomyelin

## Abstract

In eukaryotic motile cells, the active Ras (Ras-GTP)-enriched domain is generated in an asymmetric manner on the cell membrane through the excitable dynamics of an intracellular signaling network. This asymmetric Ras signaling regulates pseudopod formation for both spontaneous random migration and chemoattractant-induced directional migration. While membrane lipids, such as sphingomyelin and phosphatidylserine, contribute to Ras signaling in various cell types, whether they are involved in the Ras excitability for cell motility is unknown. Here we report that functional Ras excitability requires the normal metabolism of sphingomyelin for efficient cell motility and chemotaxis. The pharmacological blockade of sphingomyelin metabolism by an acid-sphingomyelinase inhibitor, fendiline, and other inhibitors suppressed the excitable generation of the stable Ras-GTP-enriched domain. The suppressed excitability failed to invoke enough basal motility to achieve directed migration under shallow chemoattractant gradients. The fendiline-induced defects in Ras excitability, motility and stimulation-elicited directionality were due to an accumulation of sphingomyelin on the membrane, which could be recovered by exogenous sphingomyelinase or phosphatidylserine without changing the expression of Ras. These results indicate a novel regulatory mechanism of the excitable system by membrane lipids, in which sphingomyelin metabolism provides a membrane environment to ensure Ras excitation for efficient cellular motility and chemotaxis.

## Introduction

The spontaneous generation of anterior-posterior polarity in motile eukaryotic cells, ranging from the lower eukaryote *Dictyostelium discoideum* to human leukocytes, is one example of the self-organization phenomena in living systems, for which the common molecular components and spatiotemporal dynamics have been identified (Rickert *et al.*, 2000; Vicker, 2002; Weiner *et al.*, 2007; Devreotes *et al.*, 2017). A domain-like localization of anterior signaling molecules emerges in an asymmetric manner on the membrane of cells under constant and uniform environments, which is a prerequisite for front-promoting events such as pseudopod formation in a polarized motile cell (Weiner *et al.*, 2002; Postma *et al.*, 2003; Arai *et al.*, 2010; Yang *et al.*, 2016). Recent studies have revealed that a mechanistic basis for the domain generation is provided by an excitable system (Nishikawa *et al.*, 2014); such a system was observed originally in the action potentials of neural cells and more widely in various self-organization processes such as collective cell behaviors (Hodgkin and Huxley, 1952; Aoki *et al.*, 2017; Goldbeter, 2018). Amoeboid cells of *D. discoideum* are a popular model to study an excitable system that is utilized in cellular polarity formation and migration and have revealed that a small GTPase, Ras, is a central component responsible for the excitability (Arai *et al.*, 2010; Shibata *et al.*, 2012; Shibata *et al.*, 2013; Nishikawa *et al.*, 2014; Tanabe *et al.*, 2018; Fukushima *et al.*, 2019; Huang *et al.*, 2013; Tang *et al.*, 2014; Miao *et al.*, 2017; Li *et al.*, 2018). A domain enriched with Ras in its active form (Ras-GTP) emerges on the membrane even in immobile cells that are treated with an actin polymerization inhibitor such as latrunculin A (Fukushima *et al.*, 2019). Ras-GTP recruits and activates phosphatidylinositol-3-kinase (PI3K), leading to the production of phosphatidylinositol 3,4,5-trisphosphate (PIP3) in the domain, where Pleckstrin homology domain (PHD)-containing proteins such as protein kinase B (PKB/Akt) bind to PIP3 and thus transduce signals for front-promoting events (Parent *et al.*, 1998; Meili *et al.*, 1999; Funamoto *et al.*, 2002; Sasaki *et al.*, 2004). When the excitability of Ras is enhanced with caffeine treatment, the Ras-GTP-enriched domain exhibits traveling waves with PI3K, phosphatase and tensin homolog (PTEN), PIP3 and phosphatidylinositol 4,5-bisphosphate (PIP2) (Shibata *et al.*, 2012; Fukushima *et al.*, 2019). Since the Ras excitable network exerts its spatiotemporal dynamics on the cell membrane, membrane lipids and membrane surface charges have attracted attention as factors regulating the excitability (Miao *et al.*, 2017; Banerjee *et al.*, 2022), but much about the system remains unknown.

RasG in the excitable system of *D. discoideum* shares a primary structure similar with mammalian K-Ras, which is a signaling molecule upstream of the extracellular signal-regulated kinase (ERK)/mitogen-activated protein kinase (MAPK) pathway that controls cell proliferation (Wennerberg *et al.*, 2005; Sumita *et al.*, 2014). K-Ras signaling requires sphingomyelin (SM) metabolism (Maceyka and Spiegel, 2014; van der Hoeven *et al.*, 2013; Cho *et al.*, 2016; van der Hoeven *et al.*, 2018). Several inhibitors of acid sphingomyelinase (SMase), which degrades SM into ceramide, including fendiline, desipramine, amitriptyline and imipramine, cause an abnormal accumulation of SM on the intracellular membrane (Gorelik *et al.*, 2016). Similarly, one inhibitor (D609) and one activator (2-hydroxyoleic acid; 2-OHOA) of SM synthase, which produces SM from ceramide, lead to the accumulation of intracellular membrane-associated SM. Slightly different, inhibitors of sphingosine kinase, which produces sphingosine-1-phosphate from sphingosine, including SKI-II and L-threo-dihydrosphingosine (L-threo-DHS), cause an accumulation of SM on the cell membrane. Finally, fumonisin B1, an inhibitor of ceramide synthase, which produces ceramide from sphingosine, in the salvage pathway, and myriocin, an inhibitor of serine palmitoyl transferase in the *de novo* synthesis pathway of ceramide, reduce the cellular SM content. Irrespective of these varying effects on SM levels and distributions, all these compounds reduce phosphatidylserine (PS) in the inner leaflet of the cell membrane (Cho *et al.*, 2016; van der Hoeven *et al.*, 2018). The negative charge of PS is essential for the interaction between K-Ras and the cell membrane via the C-terminal anchor sequence, which is composed of a polybasic stretch and a prenylated CAAX motif (Plowman *et al.*, 2008; Zhou *et al.*, 2014). Recently, it was shown that the electrostatic interaction serves as a driving force to form nanoclusters of K-Ras that enable a low-noise and high-fidelity signal transmission (Prior *et al.*, 2003; Murakoshi *et al.*, 2004; Plowman *et al.*, 2008; Harding and Hancock, 2008; Kholodenko *et al.*, 2010; Zhou *et al.*, 2014; 2015; 2017; 2021). SM also contributes to form lipid microdomains, such as rafts, and accumulates certain kinds of signaling molecules to enhance the signaling efficiency (Simons and Ikonen, 1997; Sezgin *et al.*, 2017). Although research has revealed the involvement of SM metabolism in the regulation of K-Ras signaling, little is known about SM metabolism in the excitability of Ras, cell motility or chemotaxis.

In this study, we revealed that SM metabolism makes an indispensable contribution to the spontaneous generation of cellular polarity and motility and thus for chemotaxis through the regulation of Ras excitability. The same chemicals as those reported to suppress K-Ras signaling were shown to suppress Ras excitability in *D. discoideum*. Fendiline-induced defects in Ras excitability, cell motility and chemotaxis were due to an accumulation of SM on the cell membrane and a depletion of PS, at least partly. This study proposes that the Ras excitable network requires SM and PS metabolism on the cell membrane for the spontaneous signal generation underlying efficient chemotaxis in motile eukaryotic cells.

## Materials and Methods

### Cell strains and culture

*D. discoideum* wild-type strain Ax2 (dictyBase strain ID: DBS0235518) was used as the parental cell line throughout this study. The cells were cultured in HL5 medium (15.4 g glucose, 7.15 g yeast extract, 14.3 g proteose peptone No. 2, 0.485 g KH_2_PO_4_, 1.28 g Na_2_HPO_4_12H_2_O, 0.2 mg folic acid, and 0.06 mg cyanocobalamin per L) supplemented with penicillin and streptomycin at 21°C. The cells in which the plasmids were introduced were selected and maintained in the presence of G-418 (20 μg mL^–1^; Wako), Blasticidin S (10 μg mL^–1^; Invivogen) or Hygromycin B (50 μg mL^–1^; Wako). The cell strain overexpressing the green fluorescent protein (GFP)-tagged Ras binding domain (RBD) of rapidly accelerated fibrosarcoma 1 (Raf1) (RBD_Raf1_-GFP) and RasG simultaneously was maintained in the presence of 14 μg mL^–1^ G-418 and 35 μg mL^–1^ Hygromycin B to allow cell growth. Plasmids for the overexpression of RBD_Raf1_-GFP and the simultaneous overexpression of the GFP-tagged PHD of PKB/Akt (PHD_PKB/Akt_-GFP) and HaloTag^®^-tagged PTEN (PTEN-Halo) are described elsewhere (Matsuoka and Ueda, 2018; Fukushima *et al.*, 2019). A plasmid for the expression of RasG is obtained by cloning *rasG* gene into BglII and SpeI sites of pHK12hyg which is a derivative of pHK12neo (NBRP ID: G90011). Inhibitors and activators of SM metabolism were kept as stock solutions dissolved in dimethyl sulfoxide (DMSO) at –30°C and added to HL5 at the indicated final concentrations; these chemicals included fendiline hydrochloride (Santa Cruz), desipramine hydrochloride (TOCRIS), amitriptyline hydrochloride (TOCRIS), imipramine hydrochloride (Nacalai), L-DHS (Sigma), SKI-II (Echelon), D609 (Cayman), 2-OHOA (Sigma), fumonisin B1 (Cayman) and myriocin (Cayman).

### Cell preparation

**Fig. 1:**
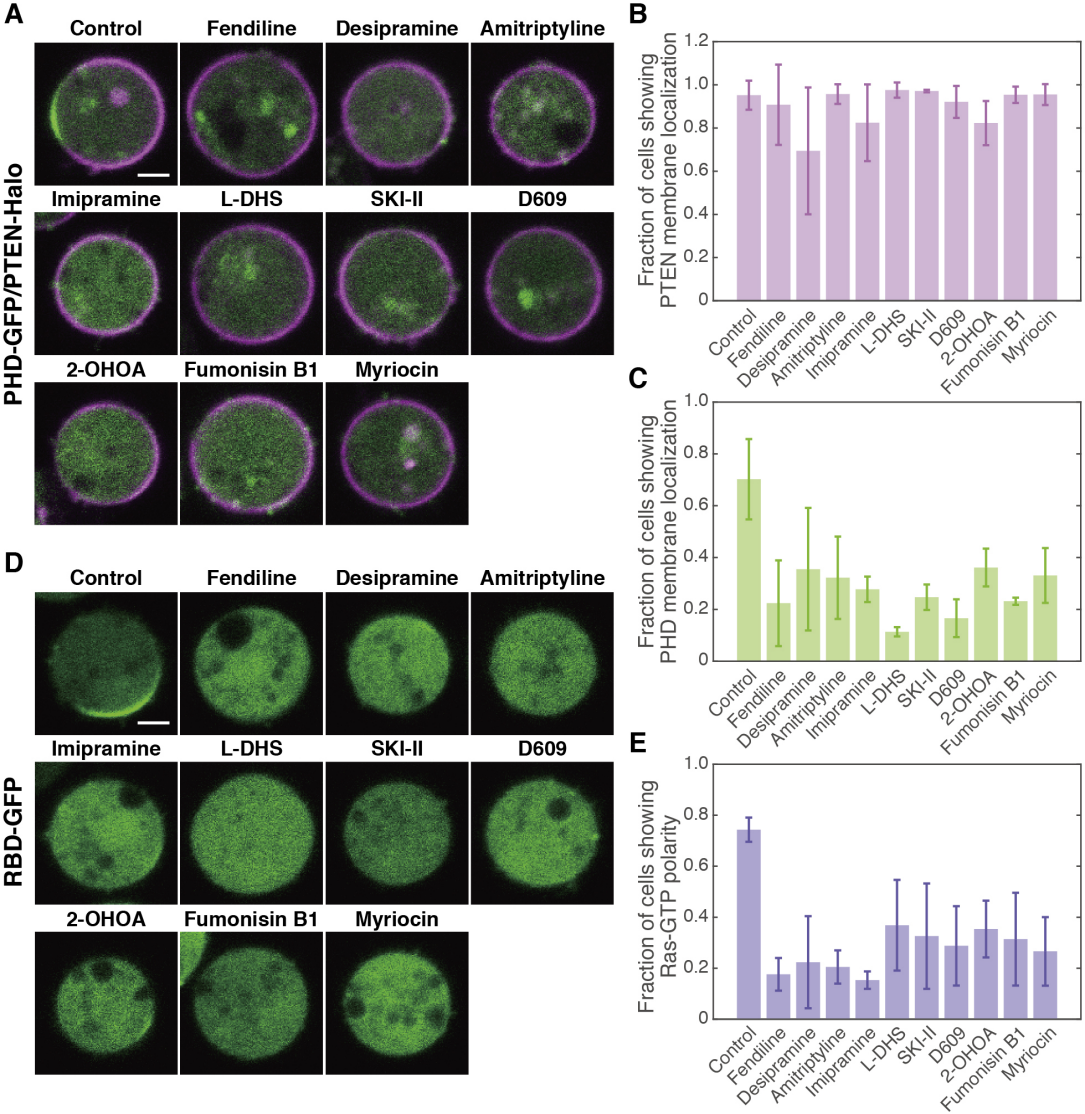
Self-organization of PIP3/PTEN and Ras-GTP polarity were suppressed by the pharmacological blockade of SM metabolism (A) Representative images of cells expressing PHD_PKB/Akt_-GFP and PTEN-Halo after drug treatment. (B) Fraction of cells showing PTEN membrane localization. (C) Fraction of cells showing PHD_PKB/Akt_ membrane localization. (D) Representative images of cells expressing RBD_Raf1_-GFP after drug treatment. (E) Fraction of cells showing Ras-GTP polarity. The cells were cultured in the presence of the indicated drug (10^–5^ M) for 48 hours and treated with 5 μM latrunculin A and 4 mM caffeine. Scale bars, 3 μm. Error bars, SD. The number of cells are listed in Table S1.

Starved cells were used throughout the study and prepared as follows. Cultured cells were washed twice with development buffer (DB; 3.5 mM KH_2_PO_4_, 1.5 mM Na_2_HPO_4_, 2 mM MgSO_4_, and 0.2 mM CaCl_2_, pH 6.5) by centrifugation (500 × *g*, 2 min) and suspended in DB at a cell density of 3 × 10^6^ cells mL^–1^. 1 mL of cell suspension was transferred to a 35-mm culture dish and kept still for 3 hours at 21°C. For the treatment with SMase during the starvation period, 1 μL SMase from *Staphylococcus aureus* (S8633; Sigma-Aldrich) was added to the 35-mm culture dish. To observe PTEN-Halo, HaloTag^®^ tetramethylrhodamine (TMR) ligand (Promega) was added to the cell suspension at a final concentration of 2 μM during the last 30 min. The starved cells were washed twice with DB by centrifugation, suspended in DB at around 5 × 10^6^ cells mL^–1^ and kept on ice until used. In Fig. 1, the cells were cultured in a 96-well glass bottom dish (Greiner) in the presence of the inhibitors and starved in the well by replacing HL5 medium with DB.

### Lysenin staining

A 100-μL cell suspension in DB containing 10 μM latrunculin A with/without fendiline and SMase was put on a coverslip and incubated for 30 min in a moist chamber. The coverslip was soaked in DB containing 4% paraformaldehyde, 0.2% glutaraldehyde and 0.1% Triton-X prechilled on ice for 5 min. The cells were further fixed with the same fixatives dissolved in DB without calcium or magnesium ions (DB(–)) for 5 min on ice. The coverslip was washed with DB(–) three times. After excess fluid was removed, the cells were covered with 100 μL DB(–) containing mCherry-Lysenin and incubated for 10 min in a moist chamber. The fluid was removed, and the coverslip was washed with DB and observed by confocal microscopy. mCherry-Lysenin was prepared according to the provider’s protocol (Abe and Kobayashi, 2017).

### Western blotting

The starved cells were suspended in DB and lysed by passing through doubled filter membranes (Whatman Nucleopore Track-etched membrane, Merck). The lysate was mixed with sample buffer, boiled for 5 min and stored at –30°C. The sample derived from 7.5 × 10^4^ cells was subjected to sodium dodecyl sulfate-polyacrylamide gel electrophoresis (SDS-PAGE) in a precast gel (SuperSep Ace 5–20%, Wako) and blotted onto a polyvinylidene difluoride (PVDF) membrane (Immobilon-P, Millipore). The membrane was treated with 5% (w/v) skim milk dissolved in Tris-buffered saline (TBS) containing 0.05% Tween-20 (TBS-T) for 1 h at room temperature (RT), washed with TBS-T, and reacted with anti-Pan-Ras mouse monoclonal antibody (OP40; Calbiochem) diluted with TBS-T at 1:2000 for 1 h at RT. The membrane was then washed with TBS-T and reacted with horseradish peroxidase-conjugated anti-mouse IgG (NA931, GE Healthcare) diluted with TBS-T at 1:5000 for 1 h at RT. After washing with TBS-T, signals were detected using ECL Prime (GE Healthcare).

### Confocal laser scanning microscopy

A cell suspension containing 10 μM latrunculin A (Sigma) and 4 mM caffeine (Wako) was placed on a coverslip of a 35-mm glass bottom dish (12-mm glass in diameter, Iwaki). The cells were allowed to settle with a 30-min incubation and then observed by confocal laser microscopy (A1, Nikon). For the removal of fendiline before the observation of RBD_Raf1_-GFP, starved cells in 200 μL suspension containing fendiline were placed on the coverslip, incubated for 30 min, washed 3 times with DB and incubated in DB without fendiline for 30 min. For cells treated with SMase or PS, a 200 μL cell suspension was placed on the coverslip, incubated for 30 min and mixed with 100 μL DB containing 0.45 μL SMase or 3 μL PS solution. PS solution was prepared as follows: 15 μL Brain PS (10 mg/mL dissolved in chloroform, 840032C, Avanti Polar Lipids) was dried under a vacuum in a glass vial to remove the solvent and redissolved in 60 μL Dulbecco’s phosphate buffered saline (14190144, Gibco, Thermo Fisher Scientific) containing 0.01% bovine serum albumin (BSA, 017-15146, Fujifilm) by sonication for 30 min. PS solution diluted with DB was centrifuged at 10,000 × *g* for 1 min, and the supernatant was added to the cells. Movies were acquired at 5-sec intervals for 30 min.

### Spatiotemporal dynamics analysis

The fluorescence intensities of RBD_Raf1_-GFP, PHD_PKB/Akt_-GFP and TMR conjugated to PTEN-Halo in an arbitrary ROI in the confocal images were measured using Fiji.

### Cell motility imaging

A 600-μL cell suspension was placed on a coverslip set in an Attofluor^TM^ cell chamber. The cells were allowed to adhere to the surface with a 20-min incubation. DB was replaced with new DB to discard unattached cells. After another incubation for 10 min, the cells were observed under an inverted microscope (IX71, Olympus) equipped with a time-lapse camera (DS-2MBW, Nikon). For the removal of fendiline, starved cells in 600 μL suspension containing fendiline were placed on the coverslip, incubated for 30 min, washed 3 times with DB and incubated in 600 μL DB without fendiline for 30 min. For the treatment with SMase or PS, starved cells in 600-μL suspension containing fendiline were placed on the coverslip, incubated for 30 min and incubated in 600 μL DB with fendiline containing 0.9 μL SMase or 6 μL PS solution for 30 min. A concentration gradient of cAMP was applied with a glass micropipette using FemtoJet (Femtotips II, Eppendorf). DB containing 100 nM cAMP filled in the glass micropipette was released at 50 hPa. The images were acquired at 5-sec intervals for 30 min.

### Cell motility analysis

The *x*- and *y*-coordinates of a cell were determined automatically using the Fiji plugin TrackMate to generate a migration trajectory (Tinevez *et al.*, 2017). The net displacement over 30 min was used to calculate the migration speed of the cell.

## Results

### Self-organization of anterior-posterior polarity required sphingomyelin metabolism

We investigated the involvement of SM metabolism in the generation of anterior-posterior polarity in *D. discoideum* cells. The distributions of PIP3 and PTEN, anterior and posterior signaling molecules, respectively, were examined in cells expressing PHD_PKB/Akt_-GFP and TMR-labeled PTEN-Halo after cultivation in the presence of SM metabolism inhibitors or activators (Funamoto *et al.*, 2002; Iijima and Devreotes, 2002; Matsuoka and Ueda, 2018). The cells were treated with 5 μM latrunculin A and 4 mM caffeine to reduce the morphological anisotropy and suppress autonomous chemoattractant signaling, respectively, and observed under confocal laser scanning microscopy (Arai *et al.*, 2010). The spontaneous generation of PIP3/PTEN polarity was observed in most (70 ± 15%) cells cultured in control medium containing DMSO, in which PIP3-enriched and PTEN-enriched domains existed in a mutually exclusive manner on the cell membrane (Fig. 1A). When the cells were cultured in the presence of the drugs, the fraction of cells showing PIP3/PTEN polarity was reduced dose-dependently and temporally (Fig. S1). In these cells, the membrane localization of PHD_PKB/Akt_ but not of PTEN was suppressed (Fig. 1B, 1C; Table S1), indicating that the drugs suppressed the signaling pathway of PI3K but not of PTEN.

The active form of RasG, which is an upstream regulator of PI3K in *D. discoideum*, can be detected by RBD_Raf1_-GFP (Sasaki *et al.*, 2004). The spontaneous generation of Ras-GTP polarity was observed in 74 ± 5% of RBD_Raf1_-GFP-expressing cells cultured in control medium, in which a Ras-GTP-enriched domain was formed on the cell membrane (Fig. 1D, 1E; Table S1). The fraction of cells showing this polarity was reduced to 18–38% after treatment with SM metabolism inhibitors. Therefore, the spontaneous generation of Ras-GTP polarity and thus PIP3/PTEN polarity requires normal SM metabolism.

### The excitability of Ras was suppressed by fendiline

**Fig. 2:**
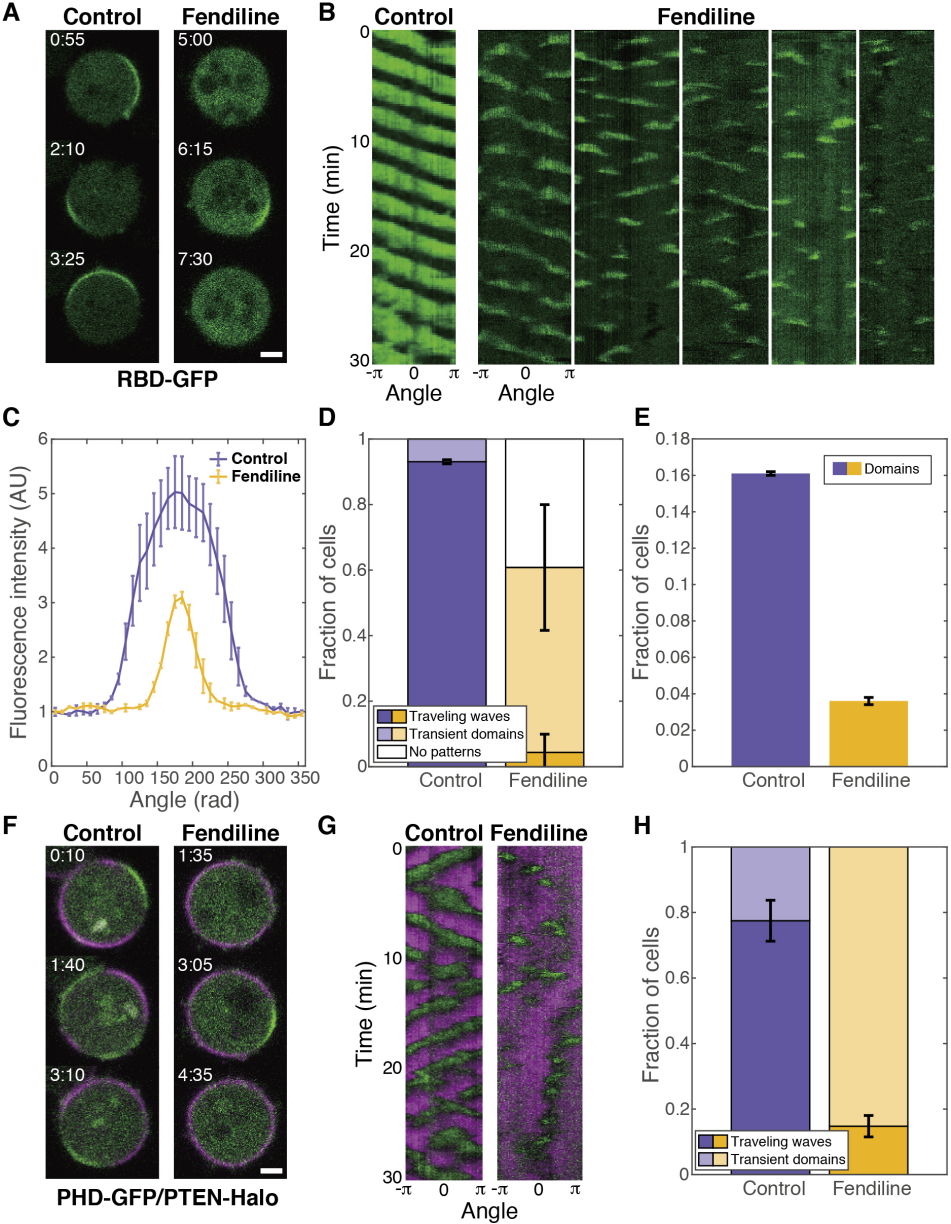
The excitability of Ras-GTP was suppressed by fendiline (A–E) The dynamics of Ras-GTP. (A) Representative time-lapse images of cells expressing RBD_Raf1_-GFP. (B) Representative kymographs of the fluorescence intensity on the cell membrane. The lifetime of the domain was 284.0 ± 313.6 sec in control cells (the mean and SD of 62 domains in 10 cells) and 62.5 ± 46.9 sec in fendiline-treated cells (the mean and SD of 179 domains in 10 cells). Note that the domain lifetime was underestimated in control cells due to the limited observation time (30 min). (C) The fluorescence intensity distribution along the cell membrane. Control, *n* = 18 cells; fendiline, *n* = 19 cells. (D) Fraction of cells showing traveling waves. Control, *n* = 72 cells; fendiline, *n* = 121 cells. (E) Fraction of cells showing transient domains in the absence of caffeine. Control, *n* = 416 cells; fendiline, *n* = 421 cells. (F–H) The dynamics of PIP3/PTEN. (F) Representative time-lapse images of cells expressing PHD_PKB/Akt_-GFP and PTEN-Halo. (G) Representative kymographs. (H) Fraction of cells showing traveling waves. Control, *n* = 120 cells; fendiline, *n* = 169 cells. Cells were treated with 5 μM latrunculin A and 4 mM caffeine unless noted otherwise. Scale bars, 3 μm. Time, min:sec. Error bars, SD.

To investigate the involvement of SM metabolism in the Ras-GTP-enriched domain in detail, fendiline, an inhibitor of acid SMase, was used hereafter (van der Hoeven *et al.*, 2013). We observed the spatiotemporal dynamics of RBD_Raf1_-GFP by confocal microscopy. The Ras-GTP-enriched domain translocated rotationally along the cell membrane as a traveling wave in control cells (Fig. 2A; Movie S1). The traveling wave was continuous for at least 30 min, as seen in the kymograph (Fig. 2B), with a mean domain size comparable to that reported previously (Fig. 2C) in 93 ± 1% of control cells (Fig. 2D). On the other hand, the traveling wave was suppressed in fendiline-treated cells (Fig. 2A; Movie S1). In this case, the Ras-GTP-enriched domain occasionally formed but disappeared usually within 1 min (Fig. 2B), and the domain size and amplitude of the Ras-GTP enrichment were smaller than in control cells (Fig. 2C). Although some cells exhibited the traveling wave continuously, the fraction of such cells was reduced to 4 ± 6% (Fig. 2D). Additionally, 56 ± 19% of fendiline-treated cells exhibited transient dynamics, and no domain was observed during the 30-min observation in 39 ± 23% of cells. The frequency of the domain formation was reduced also in the absence of caffeine, suggesting that spontaneous excitation was suppressed by fendiline (Fig. 2E) (Nishikawa *et al.*, 2014). The traveling wave of PIP3/PTEN, which is driven by the traveling wave of Ras-GTP, was also suppressed in fendiline-treated cells (Fig. 2F, 2G, 2H; Movie S1). That the traveling wave was suppressed by fendiline demonstrates that the excitability of Ras was suppressed by the drug.

### The activation of Ras was suppressed by fendiline

**Fig. 3:**
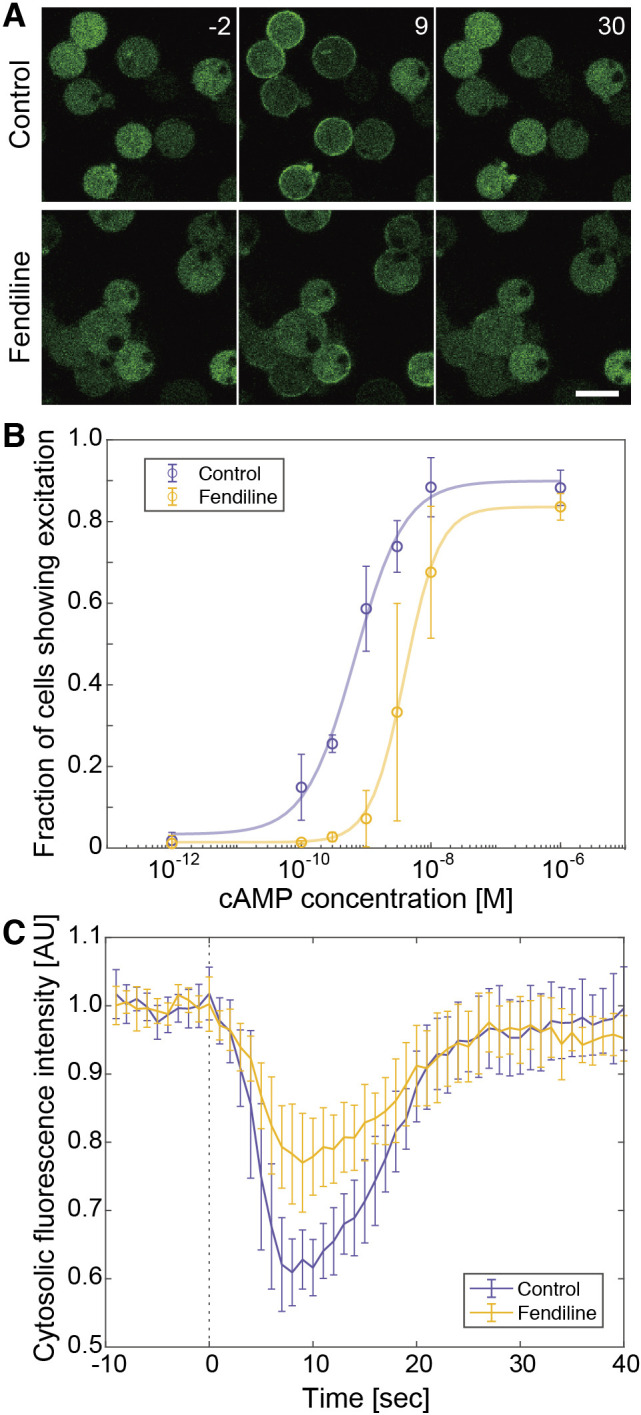
The activation of Ras was suppressed by fendiline (A) Time-lapse images of cells before and after stimulation with 3 nM cAMP onserved in the presence of 5 μM Latrunculin A. (B) Dose-dependency curves of the fraction of cells that exhibited excitation upon uniform simulation with cAMP. Data are the mean and SD of 3 independent experiments and were fitted to the equation *F*(*x*) = $min+\left(max-min\right)\frac{x^{h}}{x^{h}+K^{h}}$, with *h* = 1.1 (control, 95% confidence interval = 0.5–1.7) or 1.6 (fendiline, 95% confidence interval = 1.4–1.9). (C) The fluorescence intensity in the cytoplasm of (A). Data are the mean and SD of 10 cells that exhibited excitation. Scale bar, 3 μm. Time, sec.

To examine how the excitability was suppressed, the dynamics of excitation was analyzed by monitoring the transient membrane localization of RBD_Raf1_-GFP after the excitation was triggered with exogenous 3',5'-cyclic adenosine monophosphate (cAMP) (Fig. 3A) (Nishikawa *et al.*, 2014). The fraction of control cells that showed membrane localization increased dependently on the cAMP concentration with a half-maximum response at 0.6 nM (Fig. 3B). In fendiline-treated cells, the dose-dependent curve was shifted rightward with a half-maximum response at 4.0 nM, suggesting that the excitation occurred less effectively in these cells than in control cells. The amount of RBD_Raf1_-GFP that translocated to the cell membrane was smaller in fendiline-treated cells than in control cells (Fig. 3A): the fluorescence intensity in the cytosol was reduced by up to 39 ± 5% in control cells, but only up to 23 ± 7% in fendiline-treated cells that showed excitation (Fig. 3C). Even at the maximal response of excitation, the spatial distribution of RBD_Raf1_-GFP was heterogenous along the cell membrane in fendiline-treated cells, while RBD_Raf1_-GFP was localized on the entire surface in control cells at the maximal response. These results suggest that fendiline suppressed Ras activation, leading to the local amplification of Ras-GTP along the membrane.

### Traveling waves were not recovered by the expression of Ras

**Fig. 4:**
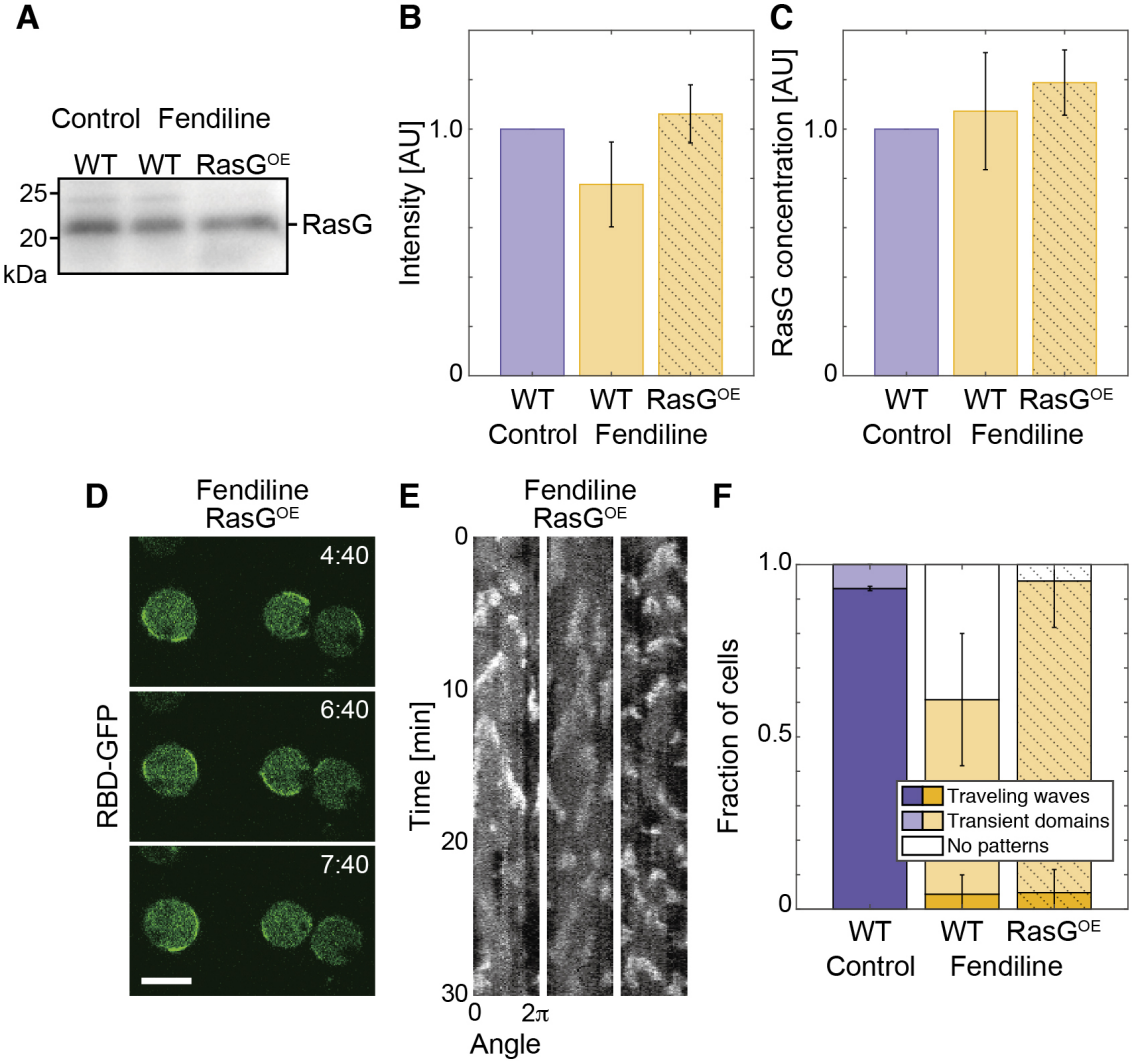
The expression of RasG was not significantly changed by fendiline and not the cause for the suppression of the traveling wave (A–C) Quantification of the expression of RasG. (A) Western blot of RasG detected with anti-Pan-Ras antibody in whole cell lysate from an equivalent numbers of cells. (B) Quantification of the band intensity in (A). (C) RasG concentration estimated by normalizing the band intensity by cell volume. The mean cell volume was estimated by assuming the cells as spheres with the radius measured in the confocal images of latrunculin A-treated cells (Fig. S2). (D–F) Dynamics of Ras-GTP in cells expressing RasG. (D) Representative images of RBD_Raf1_-GFP in cells expressing RasG. Scale bar, 10 μm. Time, min:sec. (E) Representative kymographs of RBD_Raf1_-GFP intensity measured on the cell membrane. (F) Fraction of cells showing traveling waves. The means and SDs are shown (control, WT, same as Fig. 2D; fendiline, WT, same as Fig. 2D; fendiline, RasG^OE^, *n* = 29 cells).

To test if the amount of Ras in the above experiments was insufficient for the excitation in fendiline-treated cells, the Ras expression level was examined in the cell lysate by western blotting with anti-Pan-Ras antibody, which has been known to detect RasG (Fig. 4A) (Chattwood *et al.*, 2014). Quantification of the blots derived from equal numbers of cells showed that the amount of RasG in fendiline-treated cells was approximately 78 ± 17% that of control cells (Fig. 4B). Assuming that the control and fendiline-treated cells were spheres with radii of 5.0 μm and 4.5 μm, which were the values measured in the confocal images, respectively (Fig. S2), we estimated that the concentration of RasG in individual cells was not changed significantly after the treatment (Fig. 4C). In addition, the exogenous expression of RasG did not recover the loss of traveling waves: RasG-expressing cells after cultivation with fendiline showed excitation more frequently, but the Ras-GTP-enriched domain usually disappeared rapidly and hardly propagated as a traveling wave (Fig. 4D, 4E, 4F; Movie S2). Notably, multiple domains often appeared simultaneously in these cells without further propagation (Fig. 4E). These results suggest that while the local amplification of Ras-GTP could be enhanced by an excess amount of Ras, the amount was not sufficient for spatially spreading the excited state, suggesting a mechanism other than the availability of Ras is required.

### Traveling waves were recovered by eliminating accumulated SM or supplementation with PS

**Fig. 5:**
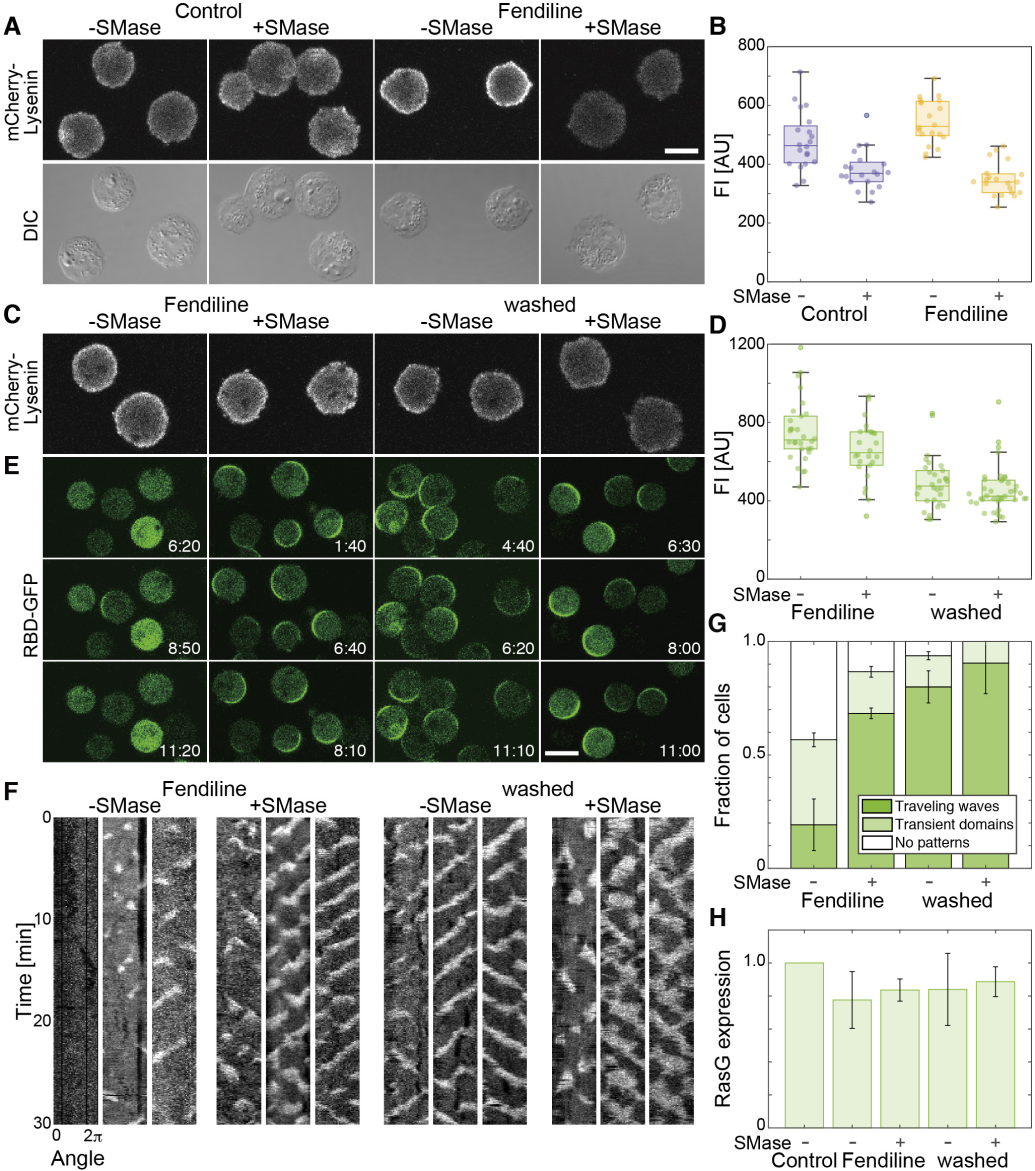
Recovery of Ras-GTP traveling waves by the elimination of SM accumulated on the cell membrane (A, B) Imaging analysis of SM levels with m-Cherry-Lysenin. (A) Representative images of mCherry-Lysenin in control (“Control”) and fendiline-treated (“Fendiline”) cells starved in the absence (“–SMase”) and presence (“+SMase”) of SMase. (B) Quantification of the fluorescence intensity on the cell membrane in (A). (C, D) Elimination of accumulated SM in fendiline-treated cells. (C) Representative images of mCherry-Lysenin in fendiline-treated cells 30 min after SMase addition (“Fendiline, +SMase”), fendiline removal (“washed, –SMase”) or both (“washed, +SMase”). (D) Quantification of the fluorescence intensity on the cell membrane in (C). (E–G) Recovery of Ras-GTP traveling waves in fendiline-treated cells. (E) Representative images of RBD_Raf1_-GFP in fendiline-treated cells 30 min after the indicated treatments. Time, min:sec. (F) Representative kymographs of RBD_Raf1_-GFP intensity measured on the cell membrane. (G) Fraction of cells showing traveling waves and transient domains. The means and SDs are shown (Fendiline, –SMase, *n* = 39 cells; Fendiline, +SMase, *n* = 32 cells; washed, –SMase, *n* = 52 cells; washed, +SMase, *n* = 37 cells). (H) Quantification results of RasG detected with anti-Pan-Ras antibody in whole cell lysates from an equivalent numbers of cells. Scale bars, 10 μm.

To test if the traveling wave was lost due to the abnormality of SM on the cell membrane, we examined the SM level by staining with Lysenin (Fig. 5A, 5B) (Yamaji *et al.*, 1998; Abe and Kobayashi, 2017). The cells were fixed with paraformaldehyde and glutaraldehyde, permeabilized with Triton X-100, and incubated with mCherry-Lysenin. In control cells that were cultivated for 48 hours in medium with DMSO and starved for 3 hours in phosphate buffer with DMSO, fluorescence was detected on the cell membrane (Fig. 5A, “Control, –SMase”). Upon treating the cells extracellularly with SMase during the starvation (Cho *et al.*, 2016), the fluorescence intensity on the cell membrane was decreased (Fig. 5A, 5B, “Control, +SMase”). This observation suggests that the amount of Lysenin reflected the SM level in *D. discoideum* cells. The intensity was higher in cells cultivated and starved in the presence of fendiline than in the absence (Fig. 5A, 5B, “Fendiline, –SMase”). The intensity was also decreased by treatment with exogenous SMase during the starvation (Fig. 5A, 5B, “Fendiline, +SMase”). Therefore, it is most likely that fendiline caused an accumulation of SM on the cell membrane via the inhibition of SMase.

Next, we examined whether reducing the amount of SM restores the Ras excitability in fendiline-treated cells. SMase addition, fendiline removal, or both were performed on cells 30 min before microscopic observation. The amount of SM quantified with mCherry-Lysenin was reduced to 51–88% after 30 min of any of these three treatments, but the degree of reduction was more pronounced with fendiline removal (“washed” in Fig. 5) than with SMase addition and was greatest with both (Fig. 5C, 5D). Concomitantly, traveling waves appeared in these cells, and the fraction of cells showing traveling waves was increased from 19% to 68–90% depending on the degree of SM reduction (Fig. 5E, 5F, 5G; Movie S3). Kymographs revealed that the spatiotemporal dynamics of the traveling waves regained in the fendiline-treated cells by the means of SM reduction were quite similar to those of control cells (Fig. 5F). Neither SMase addition nor fendiline removal affected RasG expression within the 30-min period (Fig. 5H). These results suggest that the traveling waves of Ras-GTP are suppressed due to the abnormal accumulation of SM on the cell membrane but not to decreased Ras expression.

**Fig. 6:**
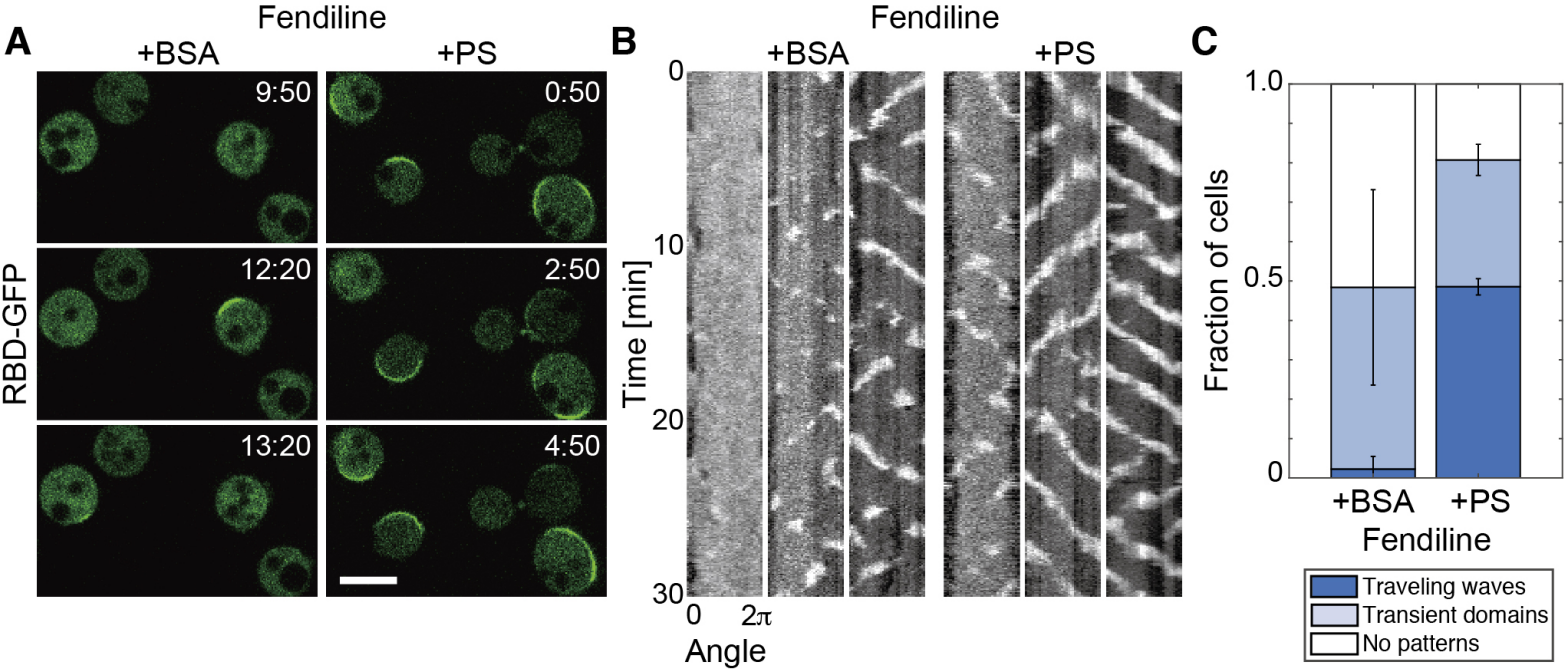
Recovery of Ras-GTP traveling waves by exogenous PS (A) Representative images of RBD_Raf1_-GFP in fendiline-treated cells incubated with BSA or PS. Scale bar, 10 μm. Time, min:sec. (B) Representative kymographs of RBD_Raf1_-GFP intensity measured on the cell membrane. (C) Fraction of cells showing traveling waves and transient domains. The means and SDs are shown (BSA, *n* = 43 cells; PS, *n* = 37 cells).

Since abnormal SM metabolism is known to cause a depletion of PS from cell membranes in mammalian cells, we examined whether exogenous PS can also recover traveling waves in fendiline-treated *D. discoideum* cells (Cho *et al.*, 2016). PS dissolved with BSA in phosphate buffer was added to the cells after 48 hours of cultivation and 3 hours of starvation in the presence of fendiline. Within 60 min after the addition, traveling waves of RBD_Raf1_-GFP appeared in 49 ± 2% of cells (Fig. 6; Movie S4). Such recovery was not observed by BSA only, suggesting that the traveling waves were triggered by PS. In addition, if cells showing transient domains are included, 81 ± 6% of fendiline-treated cells exhibited self-organization of the Ras-GTP-enriched domain after the addition of PS, but only 48 ± 28% of cells did after the addition of BSA (Fig. 6C). Attempts to monitor the PS level using the fluorescent probe Lactadherin C2 failed because this probe hardly localized to the cell membrane of *D. discoideum* cells in our experimental conditions (Kay *et al.*, 2012). Therefore, though the subcellular localization of the incorporated PS is still unclear, it is likely that PS positively regulates the excitability of Ras.

### Cell motility required sphingomyelin metabolism

**Fig. 7:**
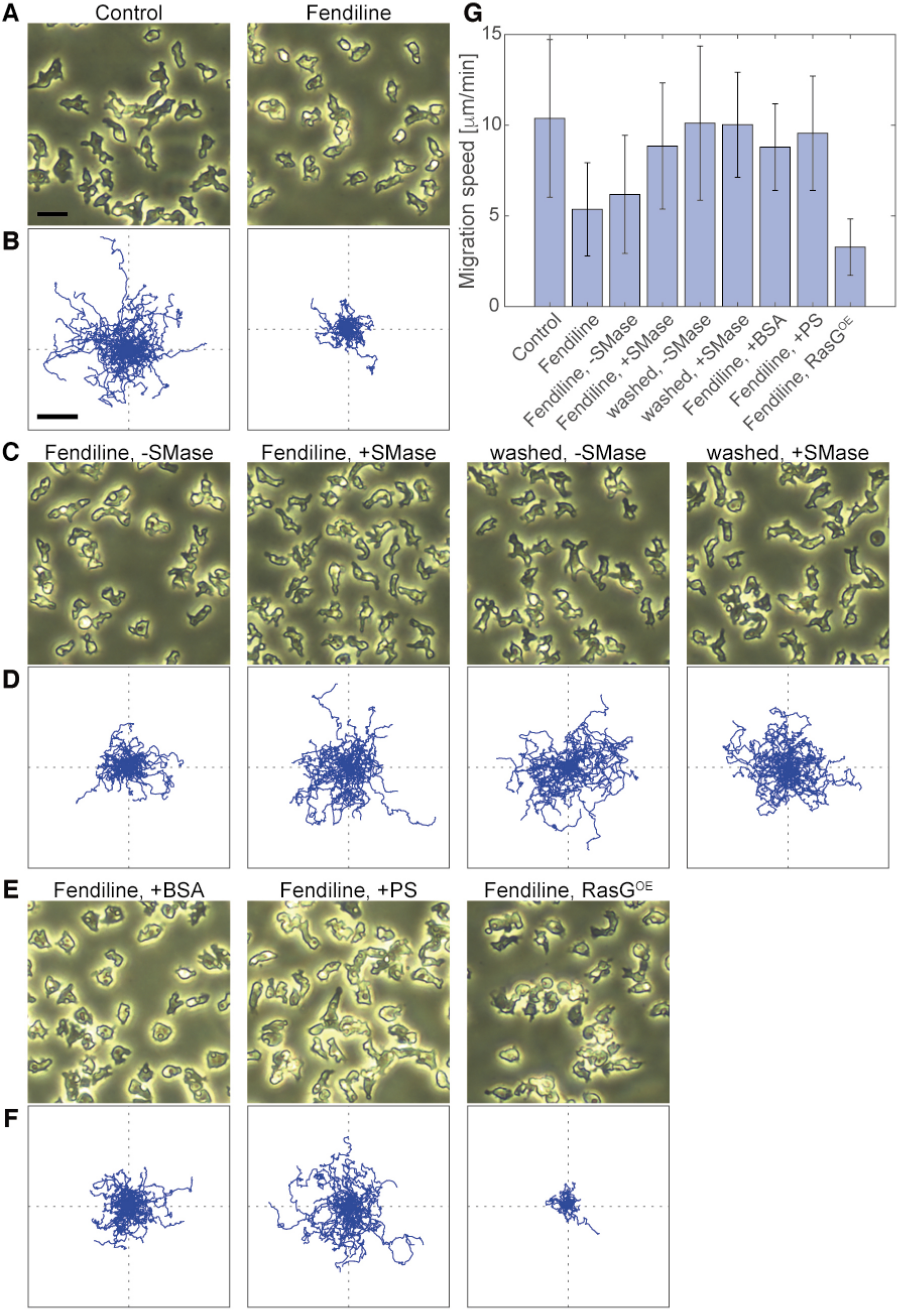
Spontaneous cell migration was suppressed by fendiline but was recovered by exogenous SMase or PS or by fendiline removal (A, C, E) Representative images of cells. Scale bar, 20 μm. (B, D, F) Migration trajectories of cells for 30 min. Scale bar, 100 μm. (G) Migration speed. The means and SDs are shown (Control, *n* = 130 cells; fendiline, *n* = 90 cells; fendiline, –SMase, *n* = 71 cells; fendiline, +SMase, *n* = 95 cells; washed, –SMase, *n* = 104 cells; washed, +SMase, *n* = 84 cells; fendiline, +BSA, *n* = 75 cells; fendiline, +PS, *n* = 73 cells; fendiline, RasG^OE^, *n* = 44 cells).

The defective Ras excitable system caused by SMase inhibition was examined in the absence of latrunculin A, a condition in which cells exhibit spontaneous cell migration (Takagi *et al.*, 2008). Cells cultivated in control media showed an elongated shape with a clear anterior-posterior morphological polarity and migrated spontaneously in random directions with an average migration speed of 11.1 ± 4.5 μm/min (*n* = 196 cells) (Fig. 7A, 7B, 7G). Cells cultivated in the presence of fendiline showed less of an elongated shape and migrated less effectively, with an average migration speed of 5.6 ± 2.8 μm/min (*n* = 113 cells). Upon SMase addition, fendiline removal or both, fendiline-treated cells recovered the morphological polarity and motility depending on the degree of the SM elimination (Fig. 7C, 7D, 7G). Recovery was also achieved with PS addition, but not with RasG expression (Fig. 7E, 7F, 7G). BSA was able to restore some motility, likely because of an RasG-independent process, such as cell-substrate adhesion signaling modulated by coating of the glass surface with BSA, but PS had an additive effect to the restoration so that the cells moved farther away from the original position compared to the BSA-added conditions. These results suggest that the Ras excitable system depends on the SM and PS metabolism for the spontaneous generation of cell polarity and motility.

**Fig. 8:**
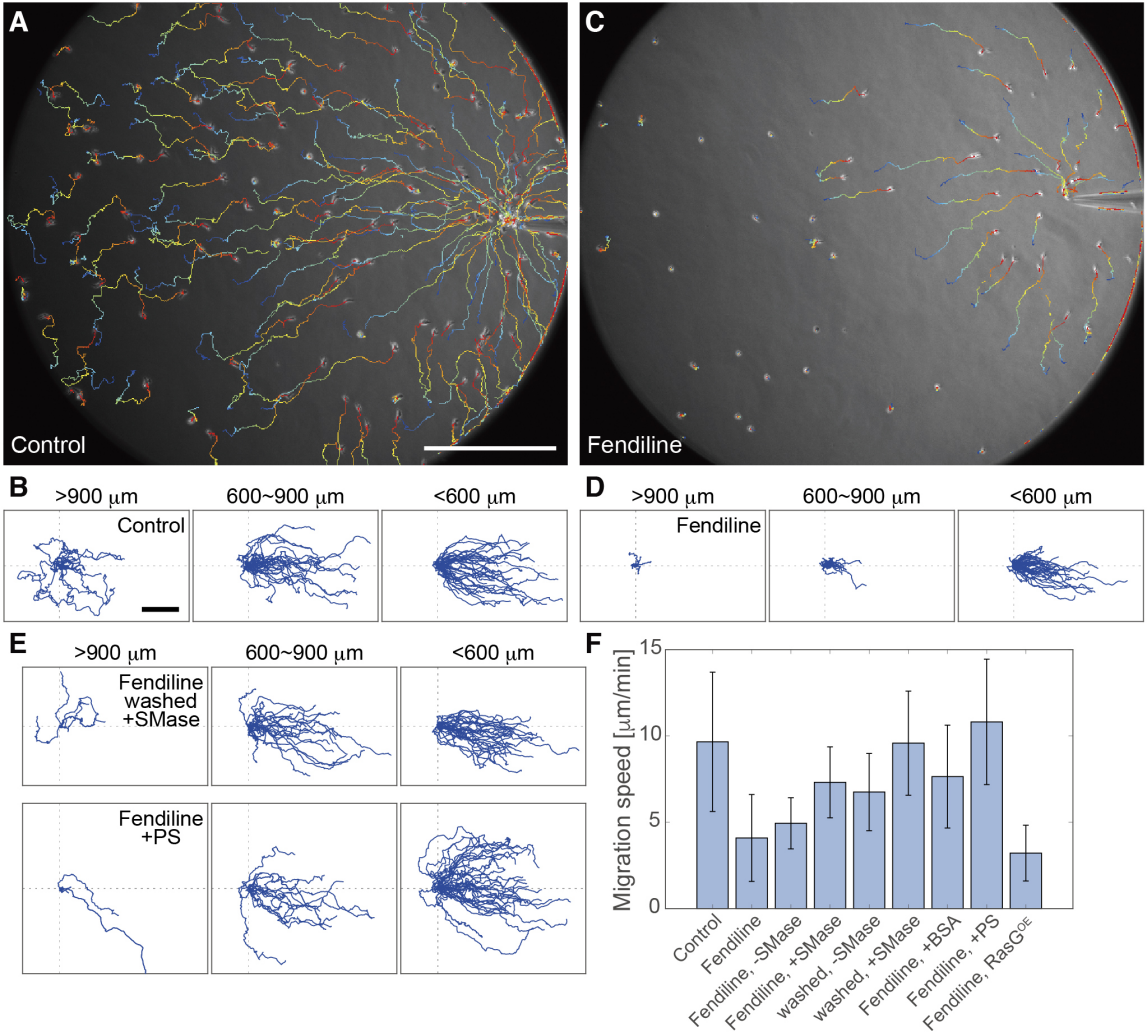
Chemotaxis under a shallow chemical gradient was suppressed by fendiline but was recovered by exogenous SMase or PS or by fendiline removal (A, C) Representative migration trajectories of control (A) and fendiline-treated cells (C) for 30 min after releasing 100 nM cAMP from a micropipette. The color code indicates the time after the gradient application, where a hotter color indicates a later time. (B, D, E) Migration trajectories for 30 min of control (B) and fendiline-treated cells (D, E). The cells were located >900 (left), 600–900 (center) and <600 μm (right) from the micropipette tip. (F) Migration speed. The means and SDs are shown (Control, *n* = 217 cells; fendiline, *n* = 130 cells; fendiline, –SMase, *n* = 79 cells; fendiline, +SMase, *n* = 79 cells; washed, –SMase, *n* = 57 cells; washed, +SMase, *n* = 98 cells; fendiline, +BSA, *n* = 104 cells; fendiline, +PS, *n* = 94 cells; fendiline, RasG^OE^, *n* = 80 cells). Scale bars, 100 μm.

We next quantitatively analyzed the chemotaxis of *D. discoideum* cells in response to cAMP. The control cells moved chemotactically with different degrees of directedness and speed depending on their distance from the tip of the micropipette (Fig. 8A, 8B, 8F). When located far from the micropipette (>900 μm), control cells often moved in the direction opposite of the chemoattractant source but finally moved up the gradient after frequent directional changes. On the other hand, fendiline-treated cells failed to show such biased motility when located far from the micropipette (Fig. 8C, 8D, 8F). Strong cAMP stimulations with high concentrations and steep gradients near the tip of the micropipette (<600 μm) induced chemotaxis efficiently in fendiline-treated cells, consistent with cAMP-induced Ras excitation at high concentration (Fig. 3). Importantly, the defects in chemotaxis under shallow and low concentration gradients were restored by SMase addition, PS addition or fendiline removal (Fig. 8E, 8F; Fig. S3). These results suggest that under a shallow and low concentration gradient, the chemotactic motility was largely dependent on the basal motility that arose due to the Ras excitable system regulated by SM and PS on the cell membrane.

Additionally, we noticed that the cells became smaller and the doubling time was increased to 20 hours from 16 hours when the cells were cultured in the presence of fendiline (Fig. S2). Therefore, multiple cellular functions involving symmetry breaking by Ras were consistently affected by fendiline, suggesting an essential role of SM metabolism in regulating Ras excitability.

## Discussion

This study revealed that the excitable system of Ras is regulated by membrane lipid species positively by PS and negatively by SM in *D. discoideum*. The metabolism of these lipids underlies spontaneous symmetry breaking in the polarity formation, cellular spontaneous motility and chemotaxis.

The roles of SM and PS in the regulation of the Ras excitable system are discussed based on the theoretical expectations below (Shibata *et al.*, 2012; Turing, 1952; Fukushima *et al.*, 2019). In general, an excitable system can generate spontaneously a domain-like pattern in an all-or-none manner with a threshold. If a signal level in the excitable system crosses the threshold, the system reaches an excited state and returns to the resting state autonomously, generating a transient increase of the signal as an excitation. If the frequency of the excitation is somehow increased, the excitation takes place repeatedly leading to an oscillation, which sometimes causes a traveling wave through the spatial spreading of the excitation. Since the accumulation of SM suppressed the generation of the traveling wave (Fig. 2 and 5), excess SM likely suppressed the frequency with which the Ras excitable system exceeded the threshold. Considering that the PS level is controlled anti-correlatively with the SM level and it promotes the membrane localization of Ras (Cho *et al.*, 2016), we concluded that defects in the traveling wave generation by SM accumulation are due to less PS on the membrane. The observation that exogenous PS regenerates the traveling wave in SM-accumulated cells is consistent with this idea (Fig. 6). The expression of RasG was expected to enhance Ras activation on the membrane, but the observed effect was partial and not sufficient to induce the traveling wave in SM-accumulated cells (Fig. 4). This observation suggests that the SM accumulation-induced decrease of PS levels has some other effect in inhibiting travelling wave generation than a depletion of Ras from the membrane. A theoretical consideration of excitable systems suggested that traveling waves can be induced by the lateral diffusion of signals (Shibata *et al.*, 2012). If true, PS may promote the diffusion of Ras-GTP so that the locally increased signal is propagated laterally to neighboring membrane regions. Such an effect can explain why the expression of RasG alone did not restore the traveling wave.

The dynamic organization of the lipid planar bilayer of the cell membrane underlies various signaling processes in eukaryotic cells. In K-Ras signaling, the lipid species in the inner leaflet have greater influence than those in the outer leaflet. K-Ras is associated with the inner leaflet, in which prenylation and multiple basic amino acid residues at the C-terminus are critical for the hydrophobic interaction between the carbon chains and the electrostatic interaction with the hydrophilic moiety of acidic phospholipids. Based on these interactions, the membrane localization and nanocluster formation of K-Ras is promoted by PS, a major acidic phospholipid actively transported to the inner leaflet by flippases under normal conditions (Sakuragi and Nagata, 2023). On the other hand, SM is mainly localized to the outer leaflet, where it serves as a structural component constituting membrane microdomains such as rafts together with sterols. Notably, the lipid heterogeneity in one leaflet influences the other. In the case of K-Ras, the nanocluster formation occurs independently of rafts (Prior *et al.*, 2003). Therefore, PS, rather than SM, is the direct regulator of K-Ras signaling, and the primary role of SM metabolism is to regulate the PS level by unknown mechanisms (van der Hoeven *et al.*, 2013; Cho *et al.*, 2016; van der Hoeven *et al.*, 2018). Our study revealed that exogenous PS was effective at recovering the defects in Ras excitability caused by SM accumulation in *D. discoideum*. Therefore, the direct regulation of Ras signaling dynamics by PS seems conserved among eukaryotic cells.

How is the excitability enhanced by PS at the molecular level? When PS promotes the membrane localization of Ras by recruiting Ras dissolved in the cytoplasm to the cell membrane and by stabilizing the association, the Ras-GTP density on the membrane increases. When PS promotes nanocluster formation, the local Ras-GTP density increases, leading to a sparse but dense localization on the membrane. These effects of PS on RasG assist with the threshold crossing and thus enhance the excitability (Lindner *et al.*, 2004). In addition, as discussed above, PS may enhance excitability by promoting the lateral diffusion of RasG. The single-molecule imaging of various transmembrane proteins has revealed three kinds of membrane regions with different viscosities on the cell membrane of *D. discoideum* cells (Takebayashi *et al.*, 2023). These microregions can generate non-homogenous spatial point patterns of membrane proteins simply by lateral diffusion. A number of studies are showing that in multiple cell types various signaling molecules undergo spatial segregation into microdomains and nanoclusters on the cell membrane with different lipid dependence (Mollinedo and Gajate, 2020; Zhou and Hancock, 2021; Banerjee *et al.*, 2022). Although the lipid constituents specific for the three microregions have not been identified in *Dictyostelium* cells, those previous studies suggest heterogeneity in PS density on the membrane, if it exists, can modulate both the nanocluster formation of Ras to trigger excitation and the lateral diffusion of Ras to generate travelling waves. Other possibilities include the enhancement of RasG activation by PS through the recruitment of RasGEF in an electrostatic manner. How PS enhances excitation can be assessed by single-molecule localization microscopy of RasG and quantitative analysis of the effect of PS on the local density and lateral diffusion on the membrane.

In conclusion, this study demonstrates that SM and PS metabolism is essential for random cell migration as well as chemoattractant-induced directional cell migration under a shallow and low concentration gradient (Fig. 7 and 8). One of the characteristics of an excitable system is that the sensitivity of the system to an external small stimulus is related to the spontaneous excitability of the system in the absence of external stimuli. Increasing the sensitivity causes more frequent spontaneous excitation, while decreasing it has the opposite effect. Therefore, the Ras excitable system is less likely to exceed the threshold with SM accumulation, causing not only less frequent excitation without cAMP (Fig. 2) but also lower sensitivity to cAMP (Fig. 3). The cells require higher concentrations of cAMP to induce Ras excitation (Fig. 3) and to trigger the front-promoting events to migrate chemotactically (Fig. 8). Both spontaneous and chemotactic motility under shallow and low concentration gradients were recovered simultaneously by PS supplementation (Fig. 7 and 8), suggesting that Ras excitability is modulated through PS and SM metabolism (Fig. 6). Thus, the extent of excitability can vary cell to cell depending on the metabolic state. In fact, a sub-population of *Dictyostelium* cells can exhibit extremely high sensitivity to cAMP at concentrations as low as 10 pM, suggesting that the receptor occupancy of only 6 cAMP molecules/cell can induce chemotaxis (Ohtsuka *et al.*, 2021). One of the origins of the spontaneous activity of living cells may be an extremely sensitive signaling system based on the characteristics of an excitable system.

## Competing Interests

The authors declare no competing interests.

## Funding

This work was supported by funds from Japan Science and Technology Agency grant no. JPMJPR1879 to SM and JPMJCR21E1 to MU, Japan Agency for Medical Research and Development grant no. JP20gm0910001 to MU, and JSPS KAKENHI grants no. 19H00982 to MU, and no. 19H05798 to SM.

## Data Availability

All data are available in the main text or the supplementary materials. All code used in the analysis are available from the authors upon reasonable request.

## Abbreviations

PI3K: phosphatidylinositol-3-kinase
PIP3: phosphatidylinositol 3,4,5-trisphosphate
PHD: Pleckstrin homology domain
PKB: protein kinase B
PTEN: phosphatase and tensin homolog
PIP2: phosphatidylinositol 4,5-bisphosphate
ERK: extracellular signal-regulated kinase
MAPK: mitogen-activated protein kinase
SM: sphingomyelin
SMase: sphingomyelinase
PS: phosphatidylserine
RBD: Ras binding domain
Raf: rapidly accelerated fibrosarcoma
GFP: green fluorescent protein
DMSO: dimethyl sulfoxide
DB: development buffer
TMR: tetramethylrhodamine
TBS: Tris-buffered saline
BSA: bovine serum albumin

## Supplementary Material

Movie S1 Dynamics of RBD_Raf1_-GFP and of PHD_PKB/Akt_-GFP and PTEN-Halo combined. The dynamics of RBD_Raf1_-GFP (upper) and of PHD_PKB/Akt_-GFP and PTEN-Halo (lower) in control (left) and fendiline-treated (right) cells. Time, min:sec. Scale bar, 3 μm.

Movie S2 Dynamics of RBD_Raf1_-GFP in cells over-expressing RasG and cultivated in the presence of fendiline. Time, min:sec. Scale bar, 10 μm.

Movie S3 Dynamics of RBD_Raf1_-GFP in cells cultivated in the presence of fendiline without (A, C) and with SMase treatment (B, D) and without (A, B) and with washing out of fendiline (C, D) before observation. Time, min:sec. Scale bar, 10 μm.

Movie S4 Dynamics of RBD_Raf1_-GFP in cells cultivated in the presence of fendiline treated with BSA (A) or PS dissolved with BSA (B). Time, min:sec. Scale bar, 10 μm.
