# Supplementary material for: Sphingomyelin metabolism underlies Ras excitability for efficient cell migration and chemotaxis
Source: Cell Struct Funct. 2023 Jul 12;48(2):145–60. doi: 10.1247/csf.23045 (PMC11496829; doi:10.1247/csf.23045)
Supplement: Supplementary file 5 — Supplementary Materials [file csf_48_23045_5.zip › 48_23045_5.docx]

Supplementary Information for

Sphingomyelin metabolism underlies Ras excitability for efficient cell migration and chemotaxis

Da Young Shin, Hiroaki Takagi, Michio Hiroshima, Satomi Matsuoka, Masahiro Ueda

Correspondence to: [matsuoka@fbs.osaka-u.ac.jp](mailto:matsuoka@fbs.osaka-u.ac.jp,), [masahiroueda@fbs.osaka-u.ac.jp](mailto:masahiroueda@fbs.osaka-u.ac.jp)

**This file includes:**

Figures S1 to S3

Table S1

Captions for Movies S1 to S4

**Other Supplementary Materials for this manuscript include the following:**

Movies S1 to S4


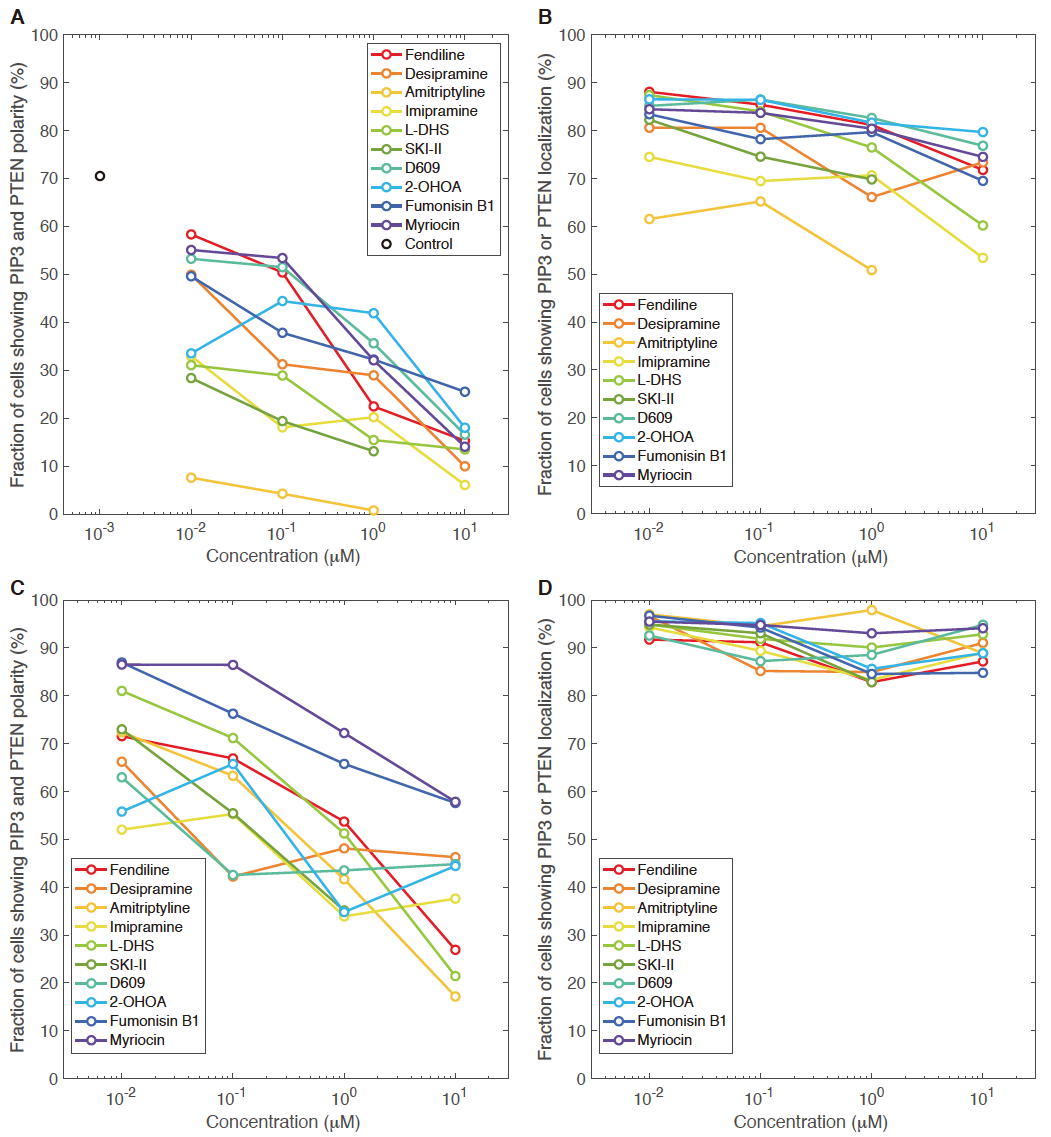


**Figure S1.**

Dose-dependent effects of inhibitors and activators of SM metabolism on the spontaneous generation of cellular polarity.

The cells were cultured in the presence of SM metabolism inhibitors and activators at the indicated concentrations for 48 hours (A, B) or 24 hours (C, D). The fraction of cells showing the membrane localization of both PHD and PTEN (A, C) and of either PHD or PTEN (B, D) are shown.


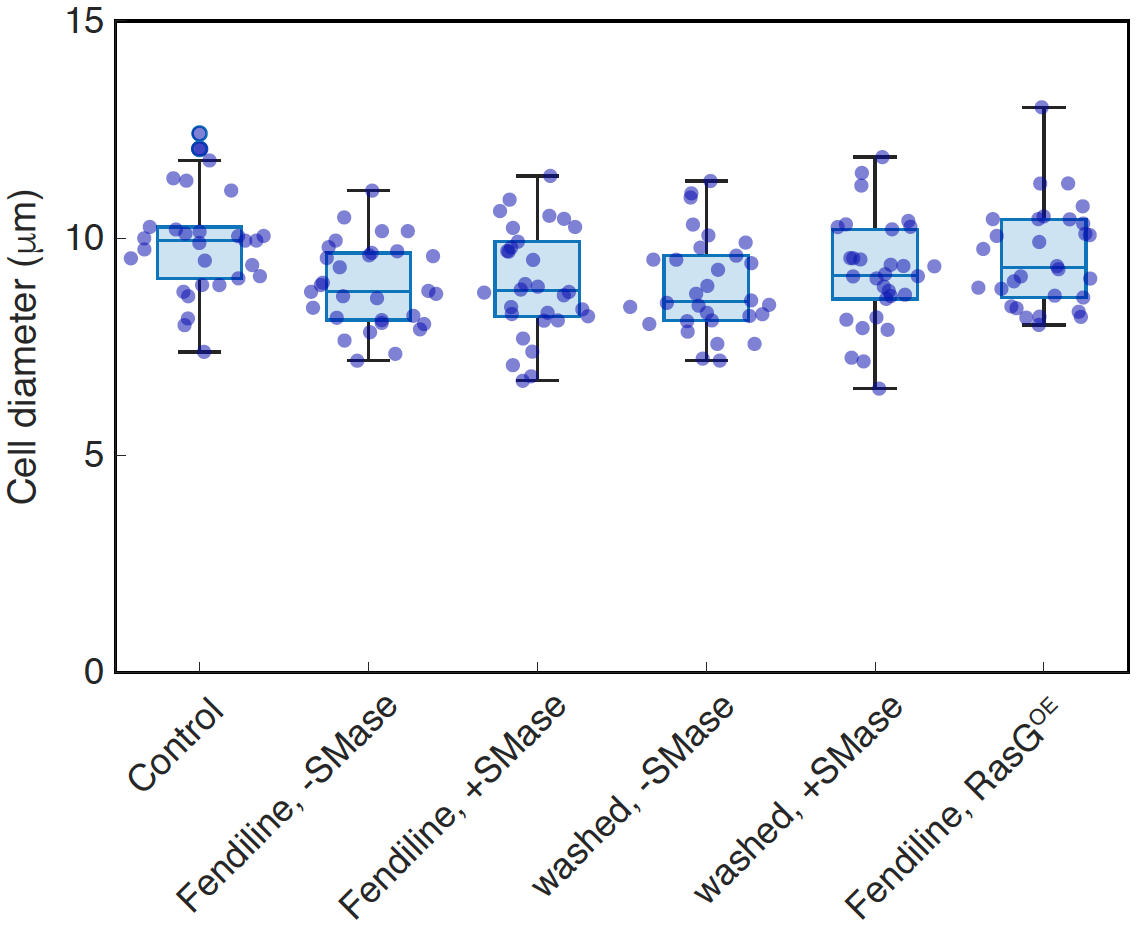


**Figure S2.**

Cell diameters.

The diameters of individual cells in the presence of latrunculin A are shown.

**
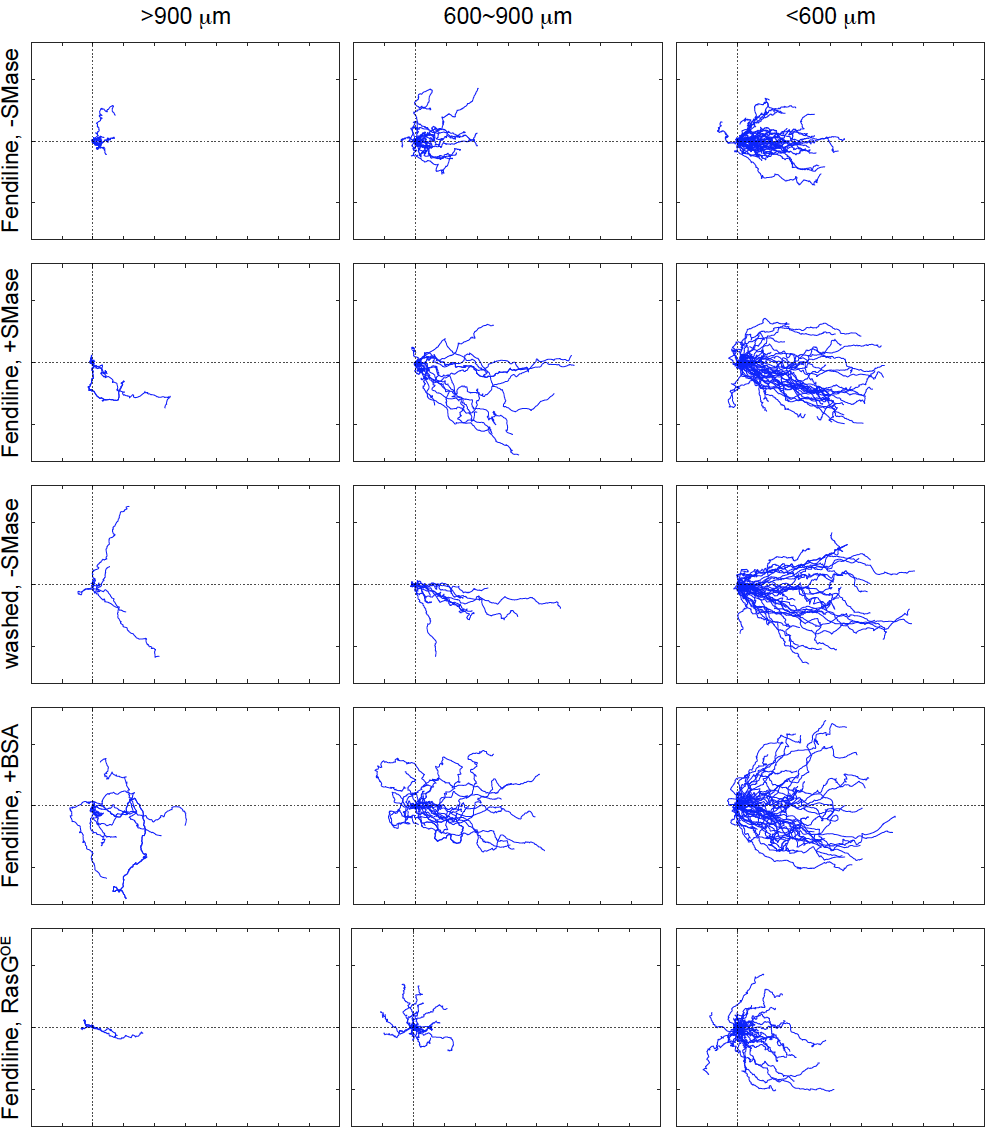
**

**Figure S3.**

Trajectories of cell migration in cAMP concentration gradients.

**Table S1.**

Data collection in Figure 1. Numbers represent number of cells.

|  | PTEN (Fig. 1B) | PIP3 (Fig. 1C) | RBD (Fig. 1E) |
| --- | --- | --- | --- |
| Control | 138 | 138 | 742 |
| Fendiline | 116 | 116 | 270 |
| Desipramine | 178 | 178 | 756 |
| Amitriptyline | 96 | 96 | 468 |
| Imipramine | 178 | 178 | 608 |
| L-DHS | 100 | 100 | 852 |
| SKI-II | 105 | 105 | 703 |
| D609 | 90 | 90 | 660 |
| 2-OHOA | 173 | 173 | 616 |
| Fumonisin B1 | 118 | 118 | 929 |
| Myriosin | 107 | 107 | 850 |

**Movie S1.**

Dynamics of RBD_Raf1_-GFP and of PHD_PKB/Akt_-GFP and PTEN-Halo combined. The dynamics of RBD_Raf1_-GFP (upper) and of PHD_PKB/Akt_-GFP and PTEN-Halo (lower) in control (left) and fendiline-treated (right) cells. Time, min:sec. Scale bar, 3 μm.

**Movie S2.**

Dynamics of RBD_Raf1_-GFP in cells over-expressing RasG and cultivated in the presence of fendiline. Time, min:sec. Scale bar, 10 μm.

**Movie S3.**

Dynamics of RBD_Raf1_-GFP in cells cultivated in the presence of fendiline without (A, C) and with SMase treatment (B, D) and without (A, B) and with washing out of fendiline (C, D) before observation. Time, min:sec. Scale bar, 10 μm.

**Movie S4.**

Dynamics of RBD_Raf1_-GFP in cells cultivated in the presence of fendiline treated with BSA (A) or PS dissolved with BSA (B). Time, min:sec. Scale bar, 10 μm.
